# Interdependent effects of fluid injection parameters on triggered aseismic slip and seismicity

**DOI:** 10.1038/s41598-022-25239-6

**Published:** 2022-12-03

**Authors:** Riddhi Mandal, Semechah K. Y. Lui

**Affiliations:** 1 Department of Chemical and Physical Sciences, University of Toronto Mississauga, Mississauga, Canada; 2 Department of Earth Sciences, University of Toronto, Toronto, Canada

**Keywords:** Solid Earth sciences, Geophysics, Seismology

## Abstract

In the context of fluid-induced seismicity, various injection parameters have been shown to affect fault behaviour differently, although existing studies about their effects sometimes show contradictory results. Aseismic slip is also known to affect seismicity, but its exact contribution remains elusive. To address these, we perform numerical modelling to understand the effects of injection volume and rate on long-term seismic and aseismic fault slip behavior. Our results suggest that both parameters can affect various aspects of fault behaviour to different extents, and, in some cases, their roles are interdependent, thus they should be examined simultaneously in order to fully characterize their effects on triggered fault responses. Within the model space, we observe the fault predominantly releasing aseismic energy, which plays a significant role in altering the timing of triggered earthquakes that follow and exhibits lasting impacts in subsequent seismic cycles. In terms of seismic responses, increasing injection rate enhances the size of the triggered cluster, while increasing injection volume increases seismicity rate of the sequence. Detailed characterization of the patterns of earthquake occurrence and moment release with respect to different injection parameters can offer insights into establishing safe bounds of injection operation and potentially mitigate seismic hazard.

## Introduction

Continental interiors are critically stressed^1^ and even small stress perturbations introduced by human activities, such as carbon storage, geothermal energy production, fracking, enhanced oil recovery and wastewater injection, can enhance seismicity^2–5^. Of all these, wastewater injection causes the biggest and most numerous earthquakes^6,7^, including several in the central US with magnitude up to $M_w$ 5.8^6^. Triggering mechanisms of injection-induced earthquakes have been proposed, namely the classic notions of the direct increase of pore pressure on the fault and indirect stress changes on the fault due to poroelastic effects^8–10^. The potential effects of injection parameters on stress perturbations in the crustal medium and the resulting triggered seismicity have therefore been major topics of interest^11–17^, although the role of each individual parameter remains elusive. Some studies have shown that injection volume is directly proportional to the amount of moment released^11,12,15^, while injection rate is found to increase seismicity^13^. On the contrary, other studies argue that seismic productivity is dependent on injection volume, with no correlation with injection rate^14^. Other parameters such as injection cycles, depth, and duration have also been shown to affect seismicity^2,16,18^.

Furthermore, more studies have demonstrated that fluid injection can also cause substantial aseismic slip, which can change the stress conditions on faults and mediate subsequent seismic activities^11,15,19–22^. For example, a propagating aseismic slip front can trigger earthquakes in weaker fault zones^23,24^, and changes in the stress field around a fault due to aseismic slip can trigger earthquakes on the same or neighbouring faults^25,26^. Both field and laboratory studies have found that most of the energy released by a fault during pore-pressure perturbation is aseismic^19,27,28^. However, it remains challenging to pinpoint the spatiotemporal extent and intensity of aseismic slip using traditional observational methods, and hence to quantify its effects.

In this study, we explore through numerical simulations how injection rate and injection volume affect both seismic and aseismic fault slip behavior. While these two parameters have been studied widely^11–15,29–32^, gaps still exist in the understanding of how they may jointly affect fault response. Assuming an isotropic and homogenous crustal medium, the volume and the rate of injection is directly proportional to the maximum pore pressure reached on the fault ($P_{max}$) and the rate of pore-pressure increase ($r_P$), respectively. Hence, we perform simulations varying $P_{max}$ and $r_P$. We employ a fully-dynamic numerical model based on the boundary integral method to simulate the long-term ($\sim$ 2400 years) evolution of slip and stress along a 60-km long rate-and-state fault under the influence of injection-induced pore-pressure perturbations^33^. Pore pressure along the fault changes with time but is considered spatially homogeneous (For details, see “Pore-pressure evolution” under “Methods”).

In our analysis, we compare and quantify the differences between simulations under pore-pressure perturbation and an unperturbed scenario, i.e., when $P_{max}$ is zero (Supplementary Fig. S1). On an unperturbed fault under regular tectonic loading, we observe an earthquake sequence with periodic events rupturing the entire seismogenic region, i.e. velocity-weakening (VW) area under the rate-and-state friction framework. The average magnitude of these seismic events is $\sim$ $M_w$ 3.6, with an average stress drop of $\sim$ 2.5 MPa. The average recurrence interval is $\sim$ 56 years. For simulations under perturbation, we assume injection occurring at a constant rate over a certain time period: Pore pressure first increases proportionally on the fault, then plateaus after pore spaces are saturated, and finally returns to the background level after injection stops (“Methods”). We systematically test a wide combinations of $P_{max}$ and $r_P$ to observe their effects on slip pattern, timings of earthquakes, and energy released over the fault. The selected model space for these two parameters reflects realistic injection wells and fault responses^34,35^: $P_{max}$ from 0.1 MPa to 2.4 MPa, which is within the bound for average stress drop on the fault, and $r_P$ from 0.001 to 1 Pa/s. Based on whether earthquakes are triggered during fluid perturbation under the applied $P_{max}$ and $r_P$ values, we divide our parameter space into categories 1 and 2, respectively.

Our study highlights that a detailed analysis of earthquake timings and moment release can offer useful insights into operational limits for injection activities. As discussed in the following sections, we find that both injection parameters display independent and interdependent effects on a wide range of fault responses, including elevated aseismic slip, clusters of triggered seismicity, and changes in the timings and magnitudes of earthquakes in multiple seismic cycles.

## Results

### Seismic response due to pore pressure change

**Figure 1:**
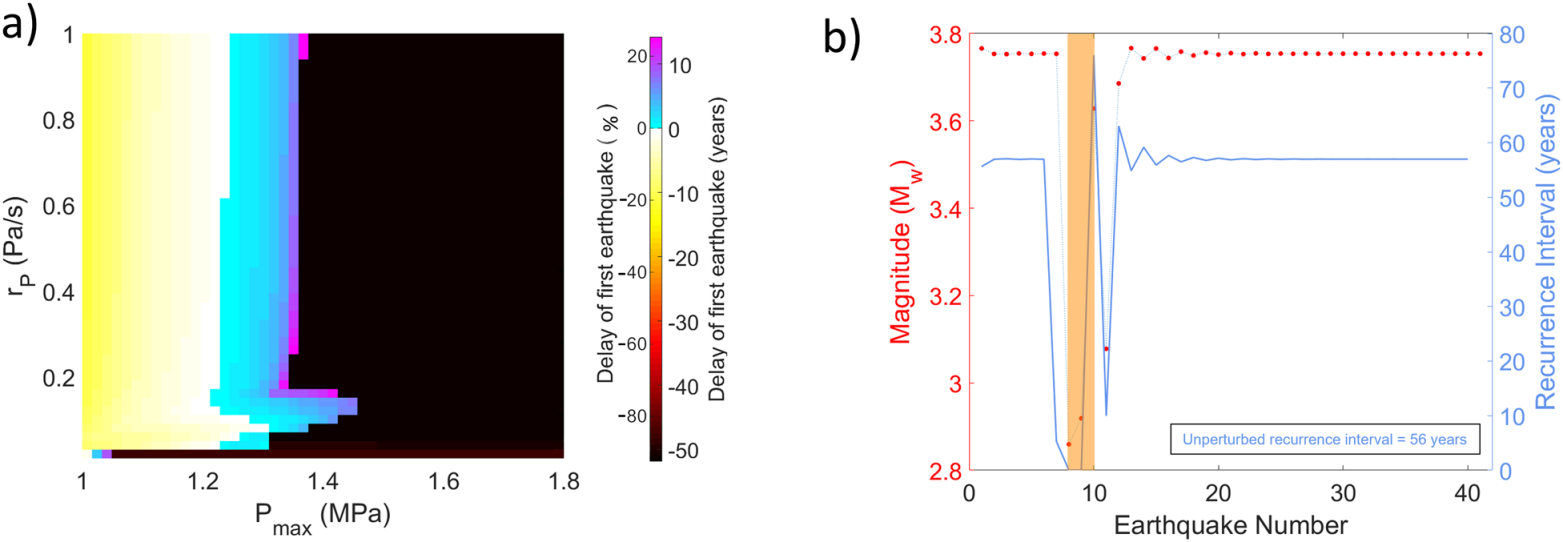
Effects of pore-pressure perturbation on earthquake timings and magnitudes. (**a**) The change in the timing of the first triggered earthquake after the start of perturbation with respect to $P_{max}$ and $r_P$, relative to an unperturbed scenario. Colour scale shown is change in timing as a percentage of an unperturbed average recurrence interval and in absolute years. Each point on the plot represents one simulation. In category 1, the first earthquake can be advanced (yellow to white) or delayed (blue to pink). In category 2, since earthquakes are triggered instantaneously, they are advanced significantly by over 80% of the seismic cycle (black to brown). (**b**) Timings and magnitudes of all earthquakes in a simulation with $r_P = 0.07$ Pa/s and $P_{max} = 1.45$ MPa. Earthquake number: ordinal number of the earthquakes that happen on the fault based on their timing. Red circle: moment magnitudes of the earthquakes. Blue line: recurrence interval, i.e. the time between two subsequent earthquakes. The orange area designates the duration over which the pore pressure on the fault was perturbed.

Under category 1, earthquakes are not triggered instantaneously during fluid injection. The applied pore-pressure perturbation stays below a certain failure threshold $P_t$ ($P_{max} < P_t$) and $P_t$ is defined as the limit separating category 1 and 2 ($P_t$ is discussed more in detail in “Influence of pore-pressure parameters on pore-pressure failure threshold”). Instead of triggered earthquakes, we observe a change in the recurrence interval of the earthquake sequence. The first earthquake that occurs after the start of perturbation can either be advanced or delayed relative to the unperturbed scenario (Fig. 1a). Interestingly, in most cases where advancement occurs, the extent of time advancement decreases as $P_{max}$ increases, and when $P_{max}$ eventually exceeds a certain value, i.e. $\sim$ 1.3 MPa in this case, the first earthquake is actually delayed compared to the unperturbed case. Overall, the timing of the first triggered earthquake has much higher dependence on $P_{max}$ than $r_P$. However, we observe interesting variations at low $r_P$ ($<0.2$ Pa/s), where the level of $P_{max}$ at which earthquake triggering transitions from time advancement to delay ($P_{max,tran}$) shows notable dependence on $r_P$ (Fig. 1a). At very low $r_P$ ($<0.1$ Pa/s), $\frac{d P_{max,tran}}{d r_P}$ is positive. For 0.1 Pa/s $< r_P < 0.2$ Pa/s, $\frac{d P_{max,tran}}{d r_P}$ is negative. Then at higher rates ($>0.2$ Pa/s), the trend is reversed again, i.e. $\frac{d P_{max,tran}}{d r_P}>0$ , although the gradient is much smaller (Fig. 1a).

**Figure 2:**
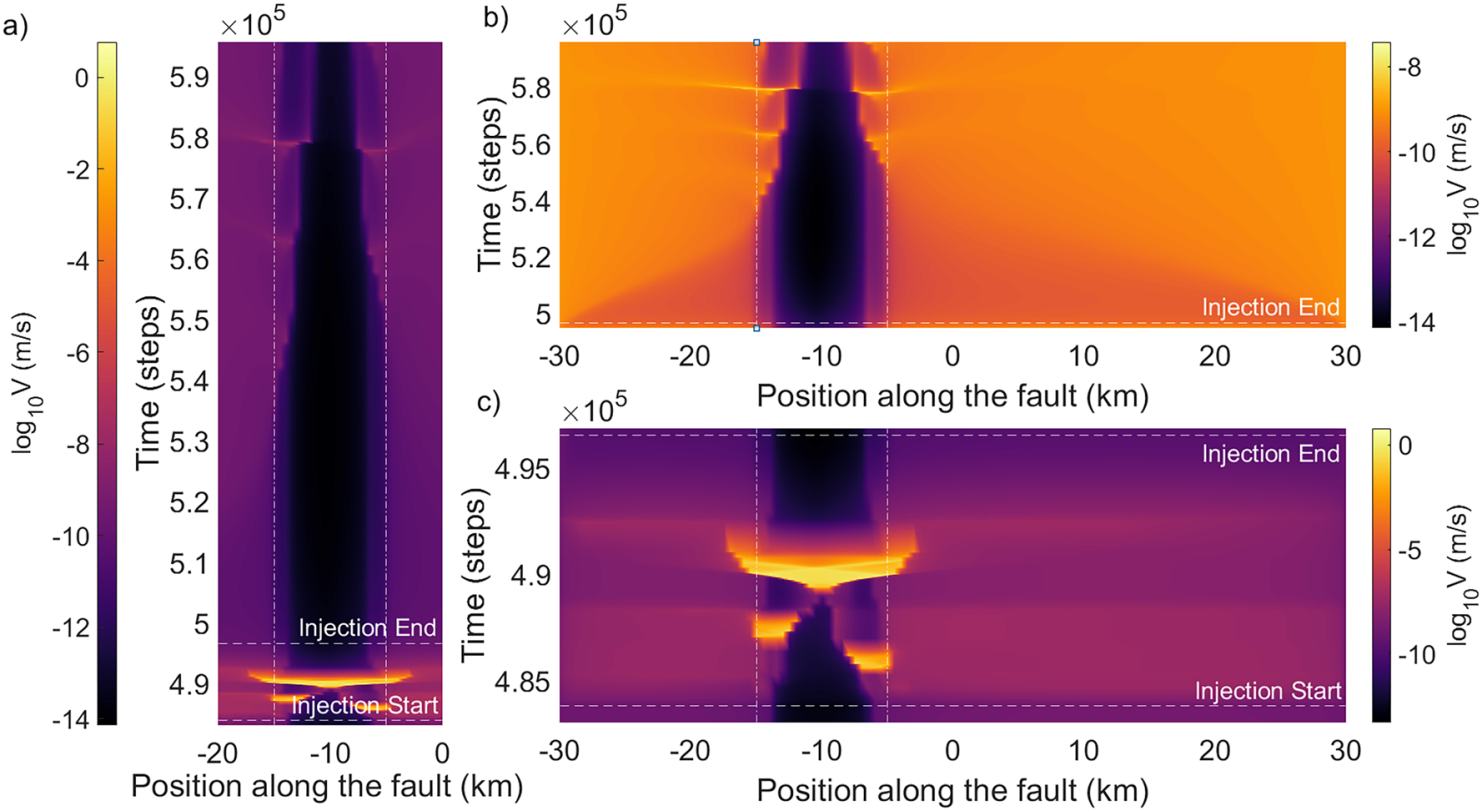
Seismic and aseismic slip response of the fault under pore-pressure perturbation. (**a**–**c**) $\log {_{10}Slip rate}$ in m/s (color scale) versus position along the fault versus time steps. Plots present results from a simulation with $P_{max} = 1.45$ MPa. Horizontal dashed lines indicate the injection start and end times. Vertical dashed lines demarcate the VW region of the fault. (**a**) Perturbation duration as well as the subsequent quiet period. (**b**) Zoomed-in figure of the interseismic period—all the slip seen in this plot is aseismic. The plot starts at the end of the injection and ends before the beginning of the next earthquake in the cycle. (**c**) Zoomed-in figure of the period under perturbation—seismic events are bright yellow in colour. Note that the color scale in (**b**) shows a narrower range of slip rate in order to highlight the aseismic transients during the interseismic period.

Under Category 2, multiple earthquakes are triggered during perturbation when $P_{max} > P_t$. These earthquakes are triggered within days to months from the start of perturbation, which is $<1\%$ of the average unperturbed recurrence interval (Fig. 1b). The average magnitude of these clusters is $\sim M_W$ 3.0, with the largest event being close to the magnitude of an unperturbed periodic earthquake. The largest triggered earthquake is usually preceded by smaller precursors which are almost one moment magnitude smaller and only rupture part of the fault (Supplementary Fig. S2). These precursors typically nucleate near the edges of the VW region and propagate inward, while the biggest earthquake usually nucleates at the center of the fault (Fig. 2a,c). As $P_{max}$ increases, the triggered earthquakes occur closer in time to each other (Supplementary Fig. S3). The triggered seismic cluster is not the sole outcome of pore-pressure perturbation. We observe significant and long-lasting changes after the perturbation ends. There is usually a seismic quiescence following the triggered cluster (Fig. 1b). The duration of this quiet period increases with increasing $P_{max}$ and ranges from 2 to 60 years longer than an unperturbed recurrence interval. Subsequent seismic cycles are also affected, with the next 7–9 earthquake cycles being either lengthened or shortened by $\sim$ 5–7 years with respect to an unperturbed scenario (Fig. 1b). The magnitudes of earthquakes on the fault also oscillate around the average unperturbed magnitude for up to 7–9 cycles (Fig. 1b). We do not find any correlation between either injection parameters and the number of perturbed cycles or the amplitude of oscillations (of timings and magnitudes).

Seismic fault responses in our simulations clearly deviate from simple predictions based on Coulomb failure model. Under the Coulomb model, an increase in pore pressure on the fault would lead to positive changes of Coulomb failure stress ($\Delta CFS$), bringing the fault closer to failure, i.e. time advancement of the next earthquake. Hence, one would not expect a time delay for the first triggered earthquake in our simulations. Also, pore-pressure perturbation in our model occurs very early in the selected interseismic period, when the overall stress state of the fault is still at a low level. The level of pore-pressure perturbation in our simulations (up to 1.8 MPa) should cause a $\Delta CFS$ far less than the average event stress drop, and is insufficient to directly bring the fault to failure. Yet we observe instantaneous earthquake triggering (Category 2). Furthermore, the Coulomb model is also unable to predict different seismic responses at changing $r_P$, as opposed to what our results show. For example, for $P_{max} = 1.2$ MPa, the first earthquake from the start of perturbation is advanced when $r_P = 0.1$ Pa/s but delayed when $r_P = 0.2$ Pa/s. Our findings have strong implication that induced earthquake triggering is potentially dominated by factors other than pore-fluid effect, such as aseismic slip, which will be further discussed in the following sections.

**Figure 3:**
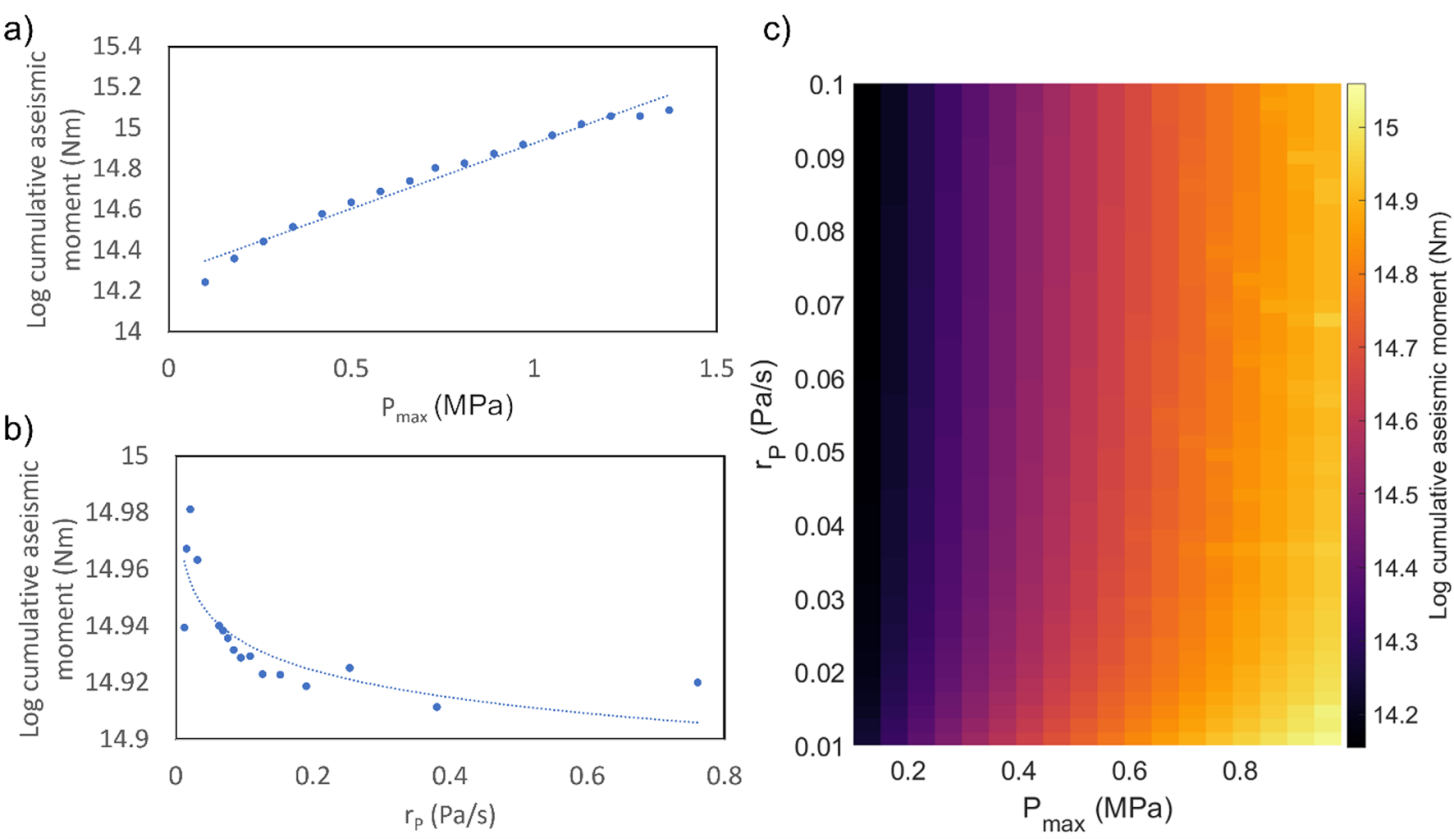
Aseismic response of the fault under pore-pressure perturbation. (**a**) Cumulative aseismic moment released during perturbation versus $P_{max}$ for category 1 at $r_P$ = 0.07 Pa/s. (**b**) Cumulative aseismic moment released during perturbation versus $r_P$ for category 1 at $P_{max}$ = 1 MPa. (**c**) Cumulative aseismic moment released during perturbation (colour scale) vs $P_{max}$ vs $r_P$.

### Aseismic fault response and its sensitivity to pore pressure parameters

Apart from changing the seismic pattern on the fault, pore-pressure perturbation also triggers and alters aseismic slip significantly, which is believed to be an important factor driving the wide range of seismic patterns as compared to the Coulomb failure model predictions. Substantial aseismic transients are observed in both Category 1 and 2 simulations.

In category 1, heightened aseismic activity is observed during perturbation. The released aseismic moment increases with increasing $P_{max}$. For instance, when $r_P$ = 0.07 Pa/s, the cumulative aseismic moment released during the perturbation increases by one order of magnitude as $P_{max}$ increases by $\sim$ 1.5 MPa (Fig. 3a). Aseismic moment releases stress and stabilizes the fault, bringing it farther from failure. Hence, with increasing $P_{max}$, we observe a gradual decreasing time advancement and eventually a time delay of the first triggered earthquake. In contrast, the released aseismic moment during perturbation decreases with increasing $r_P$, but with a much smaller gradient. At $P_{max}$ = 1 MPa, for example, the aseismic moment released during the perturbation decreases by only 1.14 times as $r_P$ increases from 0.01 to 0.8 Pa/s (Fig. 3b). Hence, even though the aseismic moment release is sensitive to both $P_{max}$ and $r_P$, for the most part of our model space, its dependence on $r_P$ is much lower than on $P_{max}$ (smaller vertical color gradient compared to the horizontal gradient in Fig. 3c).

On the other hand, in Category 2 simulations, the released aseismic moment does not show any strong trend with respect to $P_{max}$ or $r_P$. Similar to previous findings^19,27^, even with a triggered cluster of earthquakes, the moment released on the fault is predominantly aseismic ($> 99\%$) (Supplementary Fig. S4). Energy is released aseismically from both the velocity-weakening (VW) and strengthening (VS) regions of the fault. During injection, slip rate is heightened in the VS regions as well, which gradually decreases after the perturbation ends but takes years to return to the background level (Fig. 2a). Heightened aseismic slip is also seen in subsequent seismic cycles, long after the end of the perturbation and the occurrence of triggered earthquakes. These aseismic transients exhibit different spatial extents and intensities, but they have no dependence on $P_{max}$ or $r_P$ in general. These transients can extend up to 10 km into the VS zone and have slip rates over an order of magnitude higher than the background slip rate. They start at about 50% of the interseismic period, with the strongest ones happen towards the end of the cycle ($\sim 80\%$) (Fig. 2a,b).

Interestingly, the net aseismic moment released over the course of any of our simulations (both category 1 and 2), which is $\sim$ 2400 years long, does not depend on either $P_{max}$ or $r_P$ (Supplementary Fig. S5). We calculate the average aseismic moment released during the interseismic period for 40 years after the end of the perturbation, and compare that to the aseismic moment released during the same duration when no perturbation is applied. We find that the heightened aseismic moment release is compensated for by lower average aseismic moment release during the interseismic period after the perturbation ends (Supplementary Fig. S5).

### Influence of pore-pressure parameters on pore-pressure failure threshold

**Figure 4:**
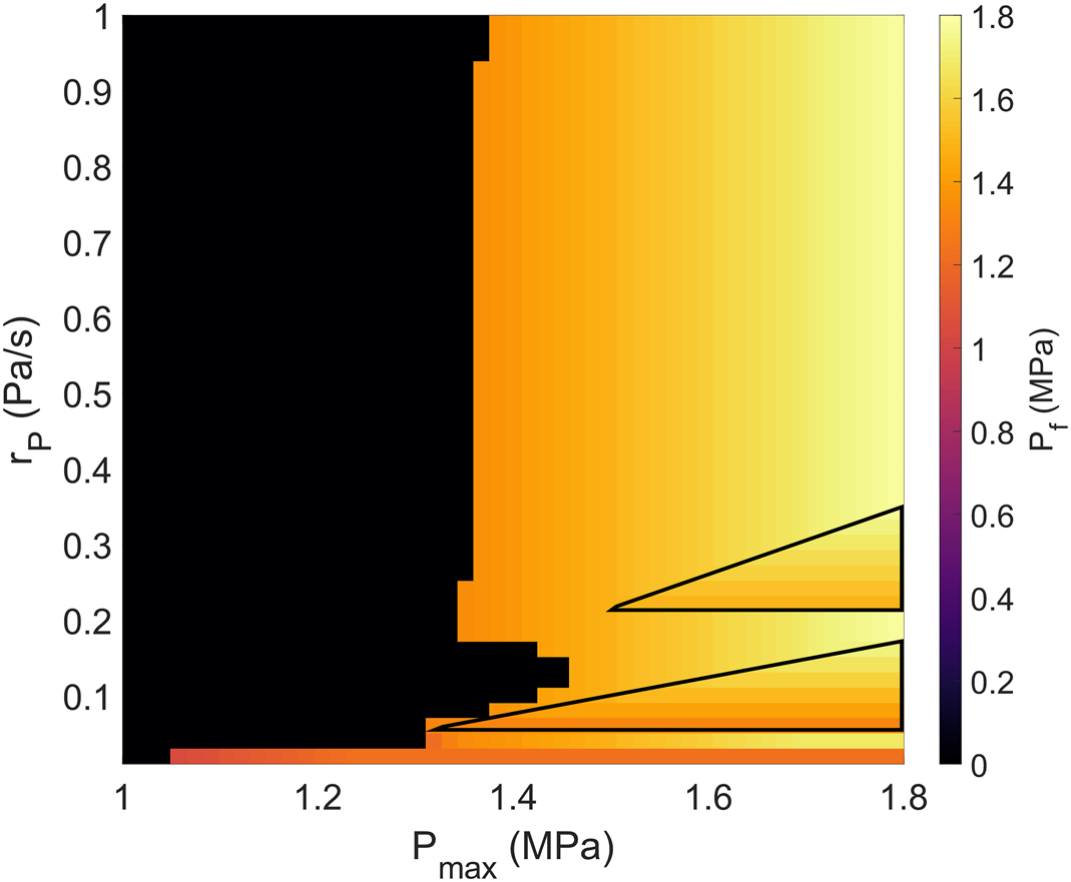
Variation of pore pressure threshold between purely aseismic and combined seismic and aseismic response on the fault. $P_t$ (color scale) vs $P_{max}$ and $r_P$. The black region is category 1, where the threshold is zero because there are no triggered earthquakes. The yellow to orange region is Category 2. For clarity, triangular boundaries have been made to highlight the regions within the model space that are dependent on $r_P$.

The observed seismic and aseismic patterns in our simulations prompt us to further examine the possible effect of injection parameters on the level of pore pressure at which the fault fails and earthquakes are triggered ($P_t$). In Category 2 simulations, $P_t$ varies between $\sim$ 50–64% of the average event stress drop on the fault. In most cases when $r_p > 0.4$ Pa/s, earthquakes are triggered shortly after pore pressure on the fault reaches $P_{max}$, so $P_t$ essentially equals $P_{max}$. Hence, $P_t$ is only sensitive to $P_{max}$ and independent of $r_P$, i.e. dominant horizontal gradient of color change from orange to bright yellow in Fig. 4. However, at lower $r_P$ ($<0.4$ Pa/s), some earthquakes are triggered before $P_{max}$ is reached, i.e. $P_t < P_{max}$. We observe interesting dependence of $P_t$ on $r_P$ in parts of our parameter space, where $P_t$ does not change smoothly with $r_P$ for a given $P_{max}$ (regions with vertical gradient of color change as marked by triangles in Fig. 4). Instead, $P_t$ first increases with increasing $r_P$, and as $r_P$ reaches a certain level, $P_t$ experiences an abrupt drop before gradually increasing again. Such dependence on $r_P$ also extends to lower $P_{max}$ when $r_P$ is at the lower bound of our parameter space (longer triangle at the bottom of Fig. 4).

**Figure 5:**
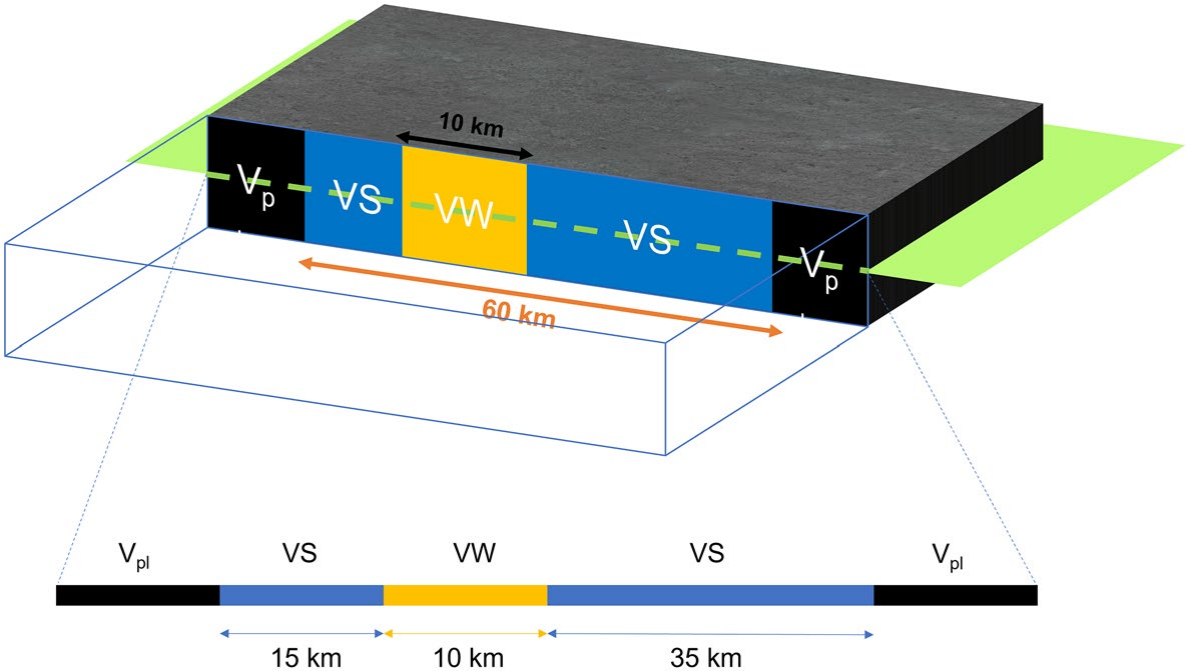
Schematic illustration of fault setup. Top: Our fault is a line fault (dashed green line) with a 60 km long VS region and a 10 km long VW region embedded in it which is offset from the center of the fault by 10 km to the left. The fault is surrounded by a bulk homogenous elastic medium. Bottom: The line fault is obtained by taking a slice of the entire medium shown by the green plane. At the edges, the fault is slipping at the background tectonic loading rate.

When examining the slip evolution on the fault, we find that the interesting dependence is due to the transition of fault slip from aseismic to seismic (Supplementary Fig. S6). For example, when $P_{max}$ = 1.7 MPa, perturbation initially triggers an aseismic transient with slip rate reaching $10^{-4}$ m/s when $r_p < 0.2$ Pa/s, preceding any induced seismic events. As $r_P$ increases, it causes the aseismic transient to increase in intensity, until a large enough region eventually nucleates at seismic slip rate and transition into a seismic event, and become the new first earthquake of the triggered cluster. Because this new earthquake now occurs earlier in time, there is a drop in the corresponding $P_t$ (Fig. 4). This indicates a positive correlation between $r_P$ and the number of earthquakes in the triggered cluster. As $r_P$ continues to increase, the rate of pore-pressure change outpaces the rate of stress response of the fault such that earthquakes are only triggered after $P_{max}$ is reached. Hence, $P_t$ stabilizes at a constant value (Fig. 4). Our results suggest that lower injection rate is prone to more unpredictable pore-pressure failure threshold due to the intricate interaction of seismic and aseismic fault slip.

## Discussion

The key general observation made from this study is that both injection volume and rate can affect various aspects of fault behaviour to different extents. While either of them may exhibit predominant effects on different fault responses, we find that their roles are also interdependent within certain part of the model space. For instance, under category 1, the amount of time advancement or delay of triggered earthquakes is primarily controlled by injection volume, but at a given volume, the extent of timing change also depends on injection rate (Fig. 1a). We also see that at low injection rate ($r_P < 0.2 Pa/s$), small changes in the rate can result in vastly different fault responses and even reversing trends (Fig. 1a). Hence, our finding suggests that, without very strong control on the rate at which the pore pressure builds up on a fault, specific observations about how injection volume affects fault response can show misleading results.

Another interesting point to note is related to the energy released on the fault. One primary observation of this study is that the energy released during perturbation is predominantly aseismic, and the cumulative aseismic moment is more sensitive to changes in injection volume than to injection rate in general, exhibiting a positive correlation. This agrees with what was suggested in McGarr and Barbour^11^, that different moment scaling relationships with respect to volume^12,36,37^ will not be necessary if the moment release accounts for both seismic and aseismic slip events. However, our finding further points out that connecting just injected volume to the moment release does not provide a full picture, since aseismic moment release also has a weak negative correlation with injection rate. The competing effects of the two parameters can lead to counter-intuitive responses. For example, a high-volume, high-rate injection may actually result in a lower amount of aseismic energy release than one with low volume and low rate (Fig. 3c). Again, such interdependent effect may be hard to observe without a big sample space for the study.

Another observation about triggered aseismic slip is its role in altering the timing of triggered seismicity. Under Category 1, the interesting change in earthquake occurrence time (from advancement to delay) implies a competing dual effect of increasing injection volume: the direct effect of the added pore pressure which brings the fault closer to failure and advances the next earthquake, as well as the increasing aseismic moment release which stabilizes the fault. Our key finding is that the effects of the latter dominate as injection volume increases. Note that in our case, since the injection is imposed early on in the seismic cycle when the state of stress is relatively low, until pore pressure reaches the failure threshold ($P_t$), triggered aseismic transients have not reached critical nucleation size and eventually subside. If injection is imposed later in the cycle, this trend may change and be more complex depending on the extent of the triggered aseismic slip relative to the critical nucleation size.

In terms of triggered seismicity, our key finding is that both injection rate and volume contribute to the pattern of earthquake triggering. Specifically, the number of triggered earthquakes is primarily controlled by the injection rate as higher rate favors dynamic slip (Supplementary Fig. S6). This observation is in agreement with other studies which suggested that higher injection rates promote dynamic slip, i.e. higher earthquake occurrence^29–32^. Another observation in our model is that triggered seismicity rate increases with injection volume (Supplementary Fig. S3). Similarly, Almakari et al. also showed that increasing maximum pore pressure along the fault can reduce the recurrence interval of the triggered sequence, i.e. enhancing seismicity rate^31^.

For computational feasibility, the several thousands of simulations in our study are performed on a 1-D fault with limited heterogeneity and no spatial variation of pore pressure. Doing this reduces the number of variables affecting the system, making it easier to characterize the effects of the injection parameters themselves. While this is advantageous for this study, these simplified assumptions may present limitations and reduce the scope of the study. Building upon the observations presented here, we work toward developing a fault model with more sophisticated spatiotemporal evolution of pore pressure and heterogeneity of fault properties to further investigate our findings. Other parameters, such as injection depth, duration and cycles, might also affect fault response. For example, Evans found that injection into the crystalline basements always produce earthquakes, suggesting a depth dependence of the effects of injection^2^. Yoon tested the effects of constant versus cyclic injection and found that cyclic injections reduce the magnitude of induced earthquakes^16^. All these additional parameters should be considered in future studies for a comprehensive understanding of fluid-induced seismicity.

In summary, by applying a state-of-the-art numerical fault model, our study explores long-term fault behavior under a suite of fluid-injection scenarios and demonstrates the importance to study injection operational parameters simultaneously, as they can exert interdependent effects on fault slip behavior such as the timing of triggered seismicity and aseismic moment release. In terms of seismic events, we find that the seismicity rate of the sequence is primarily controlled by the injection volume, while the size of the triggered cluster is controlled by the injection rate. In terms of energy release, the fault predominantly releases aseismic energy, which mostly depends on the injection volume and significantly affects the timing of subsequent triggered earthquakes. Lastly, magnitudes and timings of earthquakes remain perturbed in subsequent earthquake cycles long after injection stops. Current industrial operations often operate on a traffic light system^38,39^ without any knowledge of the safe bounds within which to operate. Action is taken only after an event occurs. Knowledge of the trend of timings of earthquakes and moment release with respect to different injection parameters can provide an estimate for safe bounds of operation and potentially mitigate seismic hazard.

## Methods

### Rate-and-state friction

The fault in our numerical model is governed by the rate-and-state friction laws, empirically derived from laboratory experiments^40–43^. The rate-and-state laws describe the dependence of fault friction on slip rate and “state” of the system. We applied the widely used Dietrich-Ruina aging law^40,44,45^:

1 $$\begin{aligned} \tau&= f(\sigma -p) \end{aligned}$$

2 $$\begin{aligned} f&= f_0+a \ln {\frac{V}{V_0}}+b\ln {\frac{V_0\theta }{L}} \end{aligned}$$

3 $$\begin{aligned} \dot{\theta }&= 1-\frac{V\theta }{L}, \end{aligned}$$

where $\tau$ is the shear stress, *f* is the frictional coefficient, $\sigma$ is the normal stress, *p* is the pore pressure on the fault, *V* is the slip rate, *L* is the characteristic slip distance, $\theta$ represents the state of the interfaces in contact, *a* and *b* are the frictional parameters, $V_0$ is the reference slip velocity, and $f_0$ is the reference coefficient of friction. When *V* is constant, $\theta$ evolves to its steady state value $\theta _{ss} = L/V$, which gives us the steady-state form of the frictional coefficient $f=f_0+(a-b) \ln {V/V_0}$. The frictional behaviour of the fault is controlled by the sign of $(a-b)$. If $(a-b)>0$, *f* increases with an increase in slip rate, thus causing steady slow slip under tectonic loading. This is known as velocity-strengthening (VS) behaviour. If $(a-b)<0$, *f* decreases with increase in slip rate, leading to fast unstable slip (i.e. earthquakes) when a large enough VW region experiences significant slip. This is known as velocity-weakening (VW) behaviour^44^. The fault in our model is made up of one VW patch embedded in a VS region. A dynamic rupture can nucleate in a VW region if the area slipping fast enough has exceeded the nucleation size $h^*$, the estimate of which in a homogenous 2-D setup is given by Refs.^46,47^:

4 $$\begin{aligned} h^*&= \frac{2}{\pi }\frac{\mu ^*bL}{(b-a)^2\sigma }, \end{aligned}$$

where $\mu ^*=\mu$ for mode III ruptures and $\mu ^* = \mu /(1-\nu )$ for mode II, $\mu$ is the shear modulus and $\nu$ is the Poisson’s ratio. This relation holds for all $a/b > 0.541$. The ratio between the length of the VW region ($D_{vw}$) and the nucleation size can thus give us some indication of how complex the fault rupture is. If $D_{vw}/h^* < 1$, earthquakes fail to nucleate and the slip is mostly aseismic, while a value greater than 1 can generate earthquakes^48,49^. Values of $D_{vw}/h^* > 6$ can cause partial rupture of the VW zone, leading to more complex behaviour^48,49^.

### Model parameters

The fault in our model is a 1-D line fault embedded in a homogenous 2-D elastic bulk medium (Fig. 5). The fault is divided into three regions—a seismogenic zone (VW region), a stable creeping zone (VS region) and a boundary zone where background loading is applied. The VW region is surrounded by VS region, with it being shifted by 10 km from the center to introduce some heterogeneity through the asymmetric loading conditions. Outside the VS zone, long-term steady slip at plate velocity $V_{pl}$ is imposed. In our models, we consider a $D_{vw}/h^*$ ratio of $\sim$6.5 to allow for partial rupture of the seismogenic zone. We classify any slip on the fault with a slip rate below $10^{-3}$ m/s as aseismic, which is slightly lower than what previous studies used (usually $10^{-1}$ m/s)^47,50^. The additional seismic slip is minimal and does not change our conclusions. Commonly used parameters are listed in Table 1.

**Table 1:** Commonly used parameters in the numerical simulations in this study.

Parameter	Symbol	Value
Shear wave speed	$c_s$	3.464 km/s
Shear modulus	$\mu$	32.04 GPa
Loading slip rate	$V_{pl}$	$10^{-9}$ m/s
Reference slip rate	$V_0$	$10^{-6}$ m/s
Reference coefficient of friction	$f_0$	0.6
Characteristic slip distance	L	0.004 m
Rate-and-state parameters in VS region	a, b	0.019, 0.015
Rate-and-state parameters in VW region	a, b	0.015, 0.019
Length of VW region	$D_{vw}$	10 km
Length of VS region	$D_{vs}$	60 km

### Pore-pressure evolution

**Figure 6:**
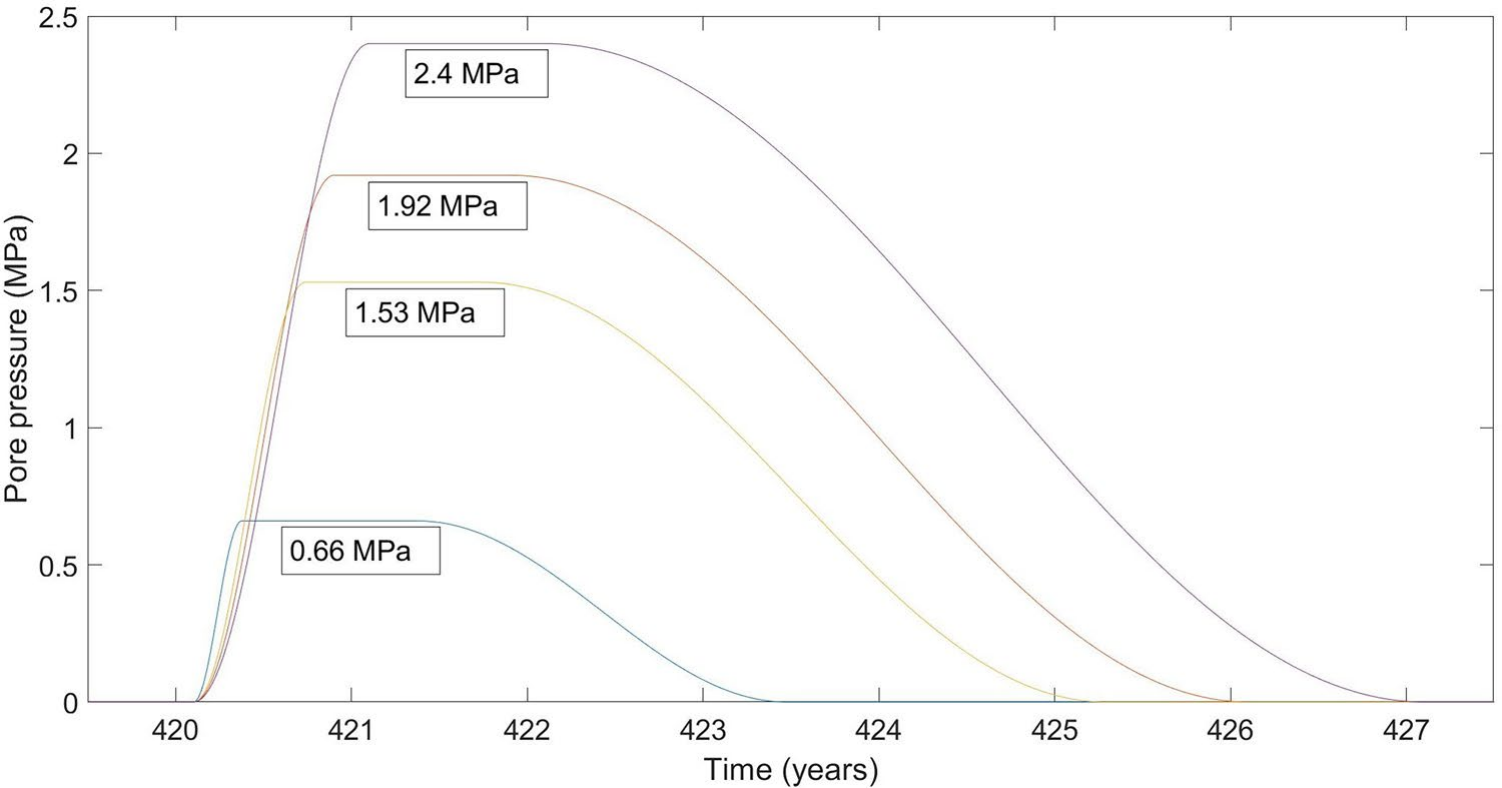
Pore pressure versus time based on Eq. 5. The average rate of increase ($p_m-p_0/t_e-t_s$) is the same in all these cases, although the instantaneous rate of increase depends on the value of the cosine function being used. In all these cases injection stops one year after the pore pressure reaches maximum.

To emulate the effects of fluid injection, we vary the pore pressure on the fault over time, while keeping it constant over space. Spatially homogenous pore pressure models have been used previously in other studies^51^. While this is a simplified assumption, spatial homogeneity can be a close approximation of certain off-fault injection scenarios. For example, if injection is located perpendicular to fault strike and at a large distance from the fault, such that the pore-pressure diffusion front reaching the fault is uniform (close to a straight line parallel to strike), pore-pressure gradient along the fault would be insignificant. In our model, a predefined time series of pore-pressure perturbation is applied on the fault. We simulate a scenario where fluid from injection operation reaches the fault and fills up the pore spaces, and thus increases the pore pressure. Once pore space is saturated, extra fluid starts diffusing out. When the rate of injection and diffusion is in equilibrium, pore pressure on the fault remains constant. After injection ends, pore pressure would decrease as fluid continues to diffuse out of the fault. At this stage, the rate of pore-pressure decrease depends solely on the permeability of the fault, which is assumed to be invariant without external forces. To emulate this process, we define a modified asymmetric Tukey window (Fig. 6) given by:

5 $$\begin{aligned} p(t) = {\left\{ \begin{array}{ll} 0 &{} t<t_s,t>t_e+t_i+\frac{p_m-p_0}{r_d} \\ p_0+\frac{p_m}{2}\left( 1-\cos {\frac{\pi (t-t_s)}{t_e-t_s}}\right) &{} t_s\le t< t_e \\ p_m &{} t_e\le t< t_e+t_i \\ p_0+\frac{p_m}{2}\left( 1+\cos {\frac{\pi (t-t_i-t_e)}{t_i+\frac{p_m-p_o}{r_d}}}\right) &{} t_e+t_i\le t < t_e+t_i+\frac{p_m-p_o}{r_d}, \end{array}\right. } \end{aligned}$$

where *p*(*t*) is the pore pressure on the fault, $p_0$ is the reference pore pressure, $t_s$ is the time when the pore-pressure front reaches the fault and pore-pressure starts increasing, $t_e$ is when the pore pressure increase and diffusion on the fault are in equilibrium, $p_m$ is the maximum pore pressure on the fault at equilibrium, $t_i$ is the time for which the equilibrium is sustained after the pore pressure stops increasing, $r_d$ is the rate at which fluid diffuses out of the fault. $r_d$ is dependent on the final permeability of the fault after injection stops and would be an intrinsic property of the fault.

We model different scenarios by keeping the rate of increase of pore-pressure constant while changing the maximum value of pore-pressure that is reached on the fault $p_m$. $r_d$ is kept constant in all the simulations at 0.6 MPa/year^52^. We also test the joint effects of rate of increase of pore pressure and maximum pore-pressure on the various observables, including slip, slip rate, and moment release. To evaluate the effects of the rate of increase more precisely, we define a pore-pressure evolution model with constant instantaneous rate of increase instead of constant average rate of increase. This is the only difference this model has from the previously defined model. It is defined as follows:

6 $$\begin{aligned} p(t)={\left\{ \begin{array}{ll} 0 &{} t<t_s,t>t_e+t_i+\frac{p_m-p_0}{r_d}\\ p_0+\frac{p_m(t-t_s)}{t_e-t_s} &{} t_s\le t< t_e\\ p_m &{} t_e\le t< t_e+t_i \\ p_0+p_m-r_d(t-t_e-t_i) &{} t_e+t_i\le t < t_e+t_i+\frac{p_m-p_o}{r_d}.\\ \end{array}\right. } \end{aligned}$$

**Figure 7:**
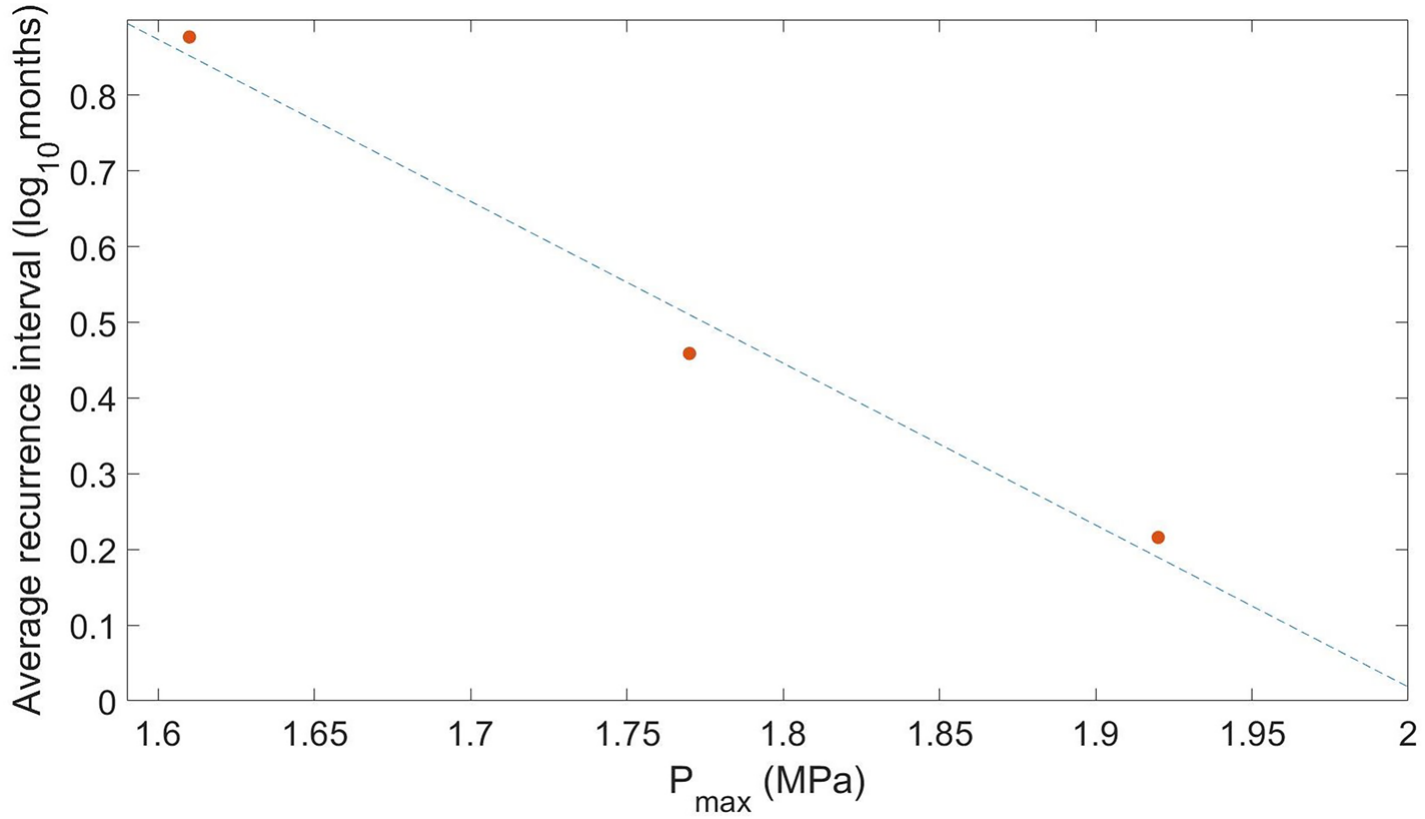
Average recurrence interval versus $P_{max}$. Average recurrence interval of triggered cluster versus $P_{max}$ for model 1 (Eq. 5). The dashed blue line shows the decreasing trend. The trend seen here is similar to that observed for model 2 (Eq. 6), as seen in Supplementary Fig. S3. This is an example of the similarity between the models. The qualitative conclusion that increasing $P_{max}$ shortens the time between the earthquakes in the triggered cluster is the same across both scenarios. Similar observations can also be made for change in $r_P$.

We model all possible scenarios with different combinations of rate of increase and max pore pressure and compare the values of the gradients of each observable to quantify the sensitivity of each observable to individual injection parameter. Both models being used have their own advantages and disadvantages, hence the need for two models. The first model provides us with a more realistic injection scenario, with no sharp changes in the pore pressure on the fault. However, during the increase in pore pressure phase, the rate of increase changes continuously. The $r_P$ in these cases refers to the average rate of change of pore pressure. For studying the sensitivity of parameters, however, knowing the average $r_P$ is not enough, as we need to fix one parameter and change the other to accurately quantify the effects of each parameter, hence the need for a model with constant $r_P$. This is more unrealistic as compared to the first model but lets us precisely control the rate of increase at any point of time. Even though the two models are different, the qualitative results mentioned in this paper do not differ between the two models (Fig. 7).

### Numerical method

Our simulations utilize the methodology of Lapusta et al. by implementing the Boundary Integral Method to model simulated earthquakes as well as relatively long interseismic periods^33^. Our method uses variable time stepping with shorter time steps during dynamic events and longer time steps during interseismic periods. This, combined with an efficient Fourier representation and the use of parallel computation, allows the simulation of long-term spontaneous fault behaviour with high spatial and temporal resolution over many seismic cycles, capturing the spatiotemporal evolution of stress and slip on the fault.

The seismic moment at any time step during the simulation is calculated by integrating slip at slip rates $10^{-3}$ m/s or higher over the VW region of the fault and multiplying by the shear modulus. We also define aseismic moment in a similar fashion, by integrating slip at slip rates less than $10^{-3}$ m/s over the VW region of the fault and multiplying by the shear modulus.

## Supplementary Information

Supplementary Information.

## Data Availability

All data are available in the main text or the supplementary materials. The corresponding author R.M. can be contacted for any further details.
