# Supplementary material for: Interdependent effects of fluid injection parameters on triggered aseismic slip and seismicity
Source: Sci Rep. 2022 Dec 3;12:20922. doi: 10.1038/s41598-022-25239-6 (PMC9719504; doi:10.1038/s41598-022-25239-6)
Supplement: Supplementary file 1 — Supplementary Information. [file 41598_2022_25239_MOESM1_ESM.pdf]

Interdependent effects of fluid injection parameters on triggered aseismic slip and seismicity

*Riddhi Mandal[1,2] and Semechah K.Y. Lui[1,2]

[1]Department of Chemical and Physical Sciences, University of Toronto Mississauga
[2]Department of Earth Sciences, University of Toronto

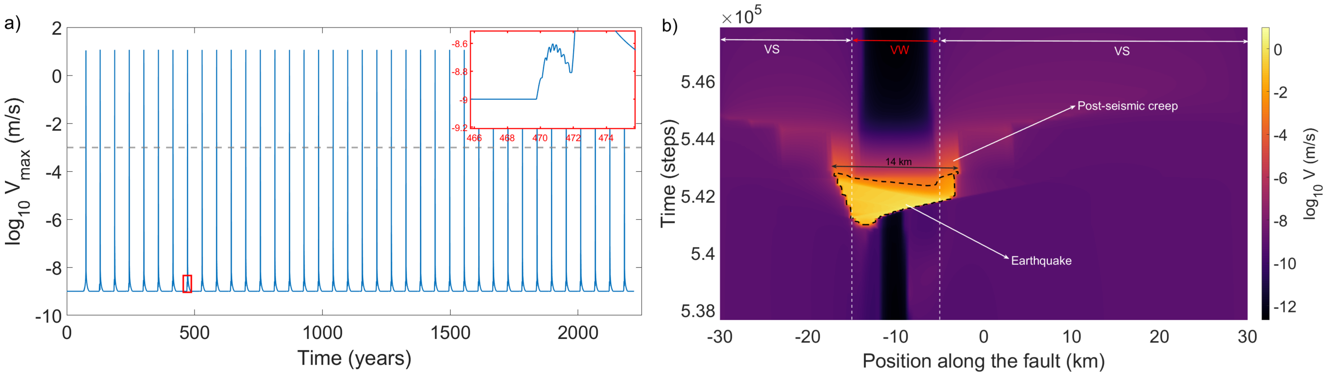

Supplementary Figure 1: Slip rate on the fault for an unperturbed scenario where P_{max}=0. a) log of maximum slip rate versus time. During interseismic periods, the highest slip rate is close to the background plate velocity (109 m/s). During dynamic events, the highest slip rate is usually recorded within the VW region and is seen as spikes on the graph. Aseismic transients, if present, show up as much smaller spikes on the graph (small red boxed region). Slip on the fault is categorized as aseismic if the slip rate is less than 103 m/s (below the dashed grey line) or seismic if it is more than 103 m/s (above the dashed grey line). (Inset) log of maximum slip rate versus time showing the details of the aseismic transient in the red box . b) Slip rate at every point on the fault versus time. This figure shows one of the earthquakes occurring on the fault. Time is presented as time steps recorded during the simulation. The bright yellow region inside the dashed black line is the earthquake. All the other colours denote aseismic slip.

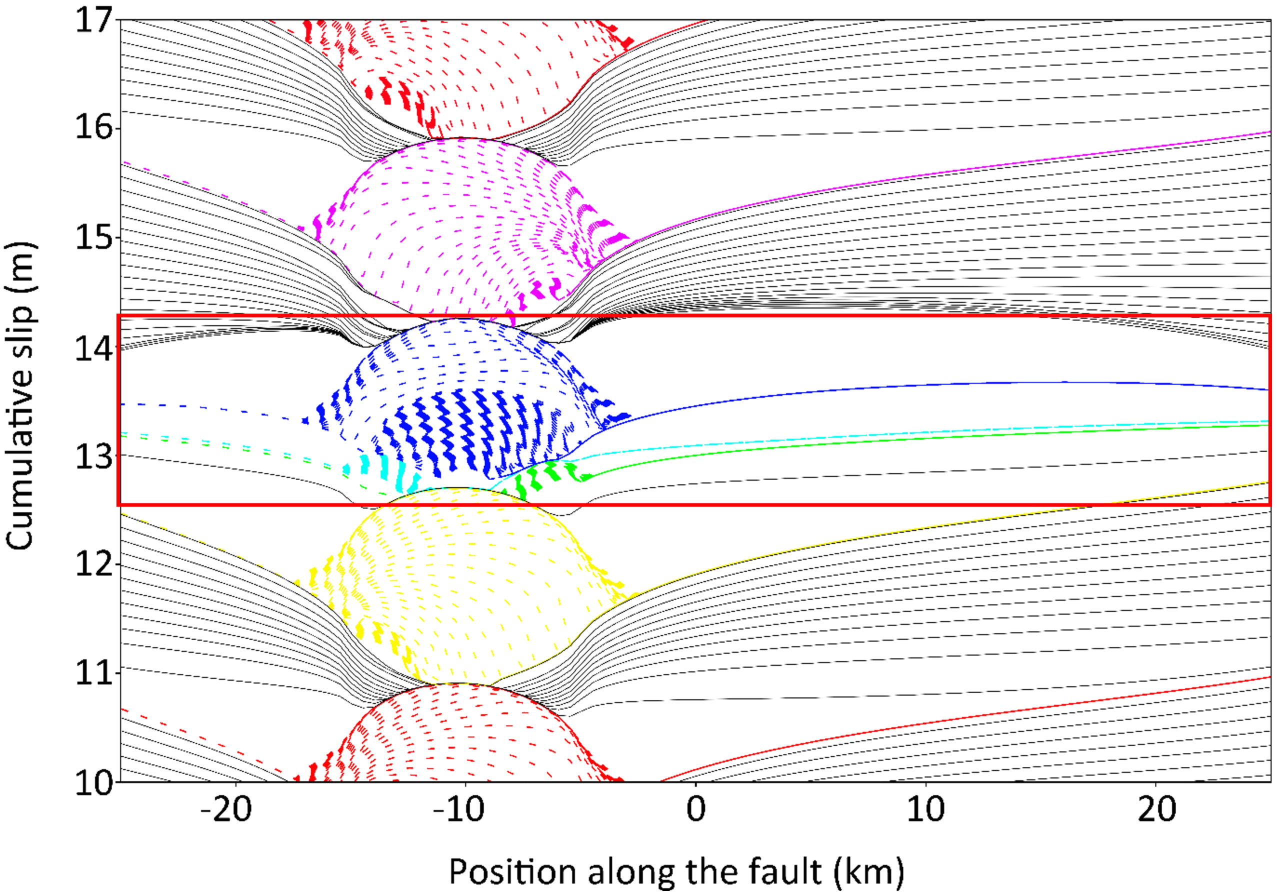

Supplementary Figure 2: Slip distribution on the fault for a triggered cluster of earthquakes. Cumulative slip on the fault versus fault position for an applied perturbation of P_{max} = 1.45 MPa and r_P = 0.07 Pa/s. Slip during an interseismic period (aseismic creep) is shown as black lines and are plotted every 5 years. The slip during an earthquake is shown as colored lines and plotted every 5 seconds. Each earthquake is represented by a different colour for easier identification of those that rupture the VW region partially. The triggered cluster is highlighted by the red box.

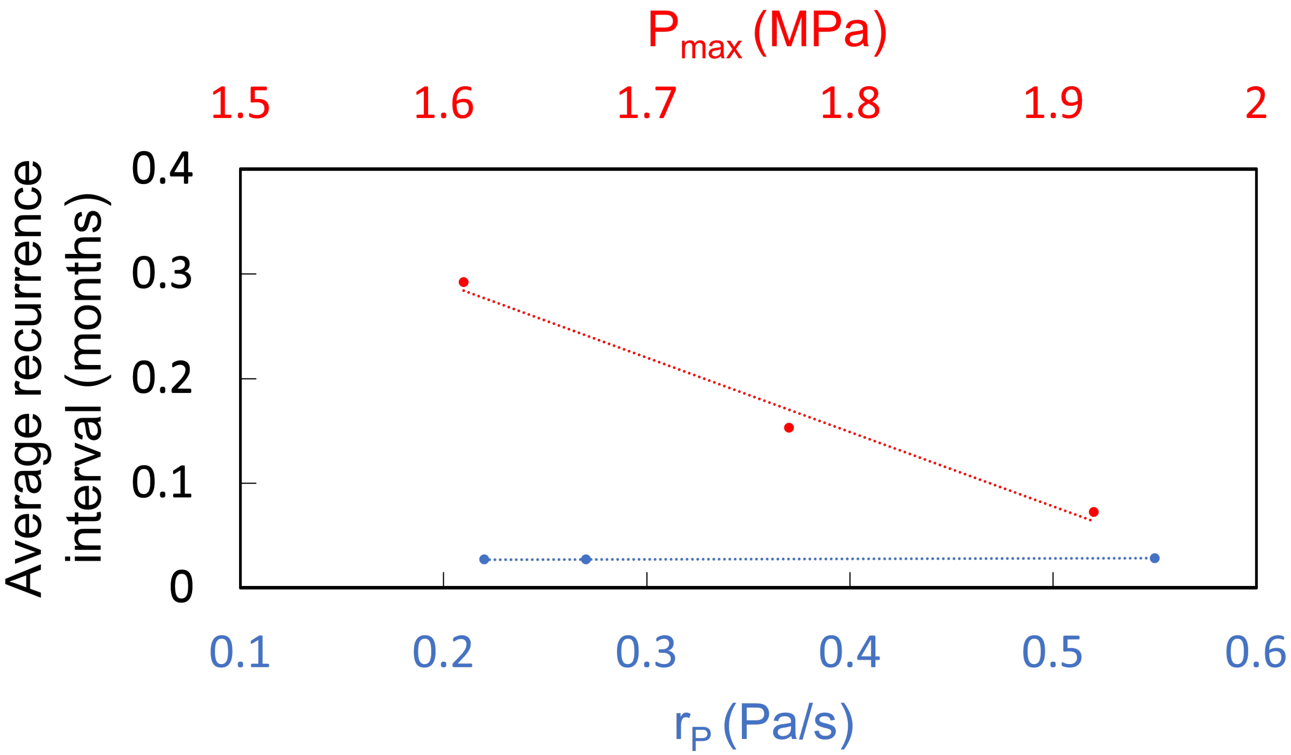

Supplementary Figure 3: Evolution of average recurrence interval as P_{max} and r_P are increased. Average recurrence interval of earthquakes in the triggered cluster versus P_{max} (red) and r_P (blue), showing the evolution of the triggered cluster. $r_P = 0.07$ Pa/s when P_{max} is varied and $P_{max} = 1.7$ when r_P is varied.

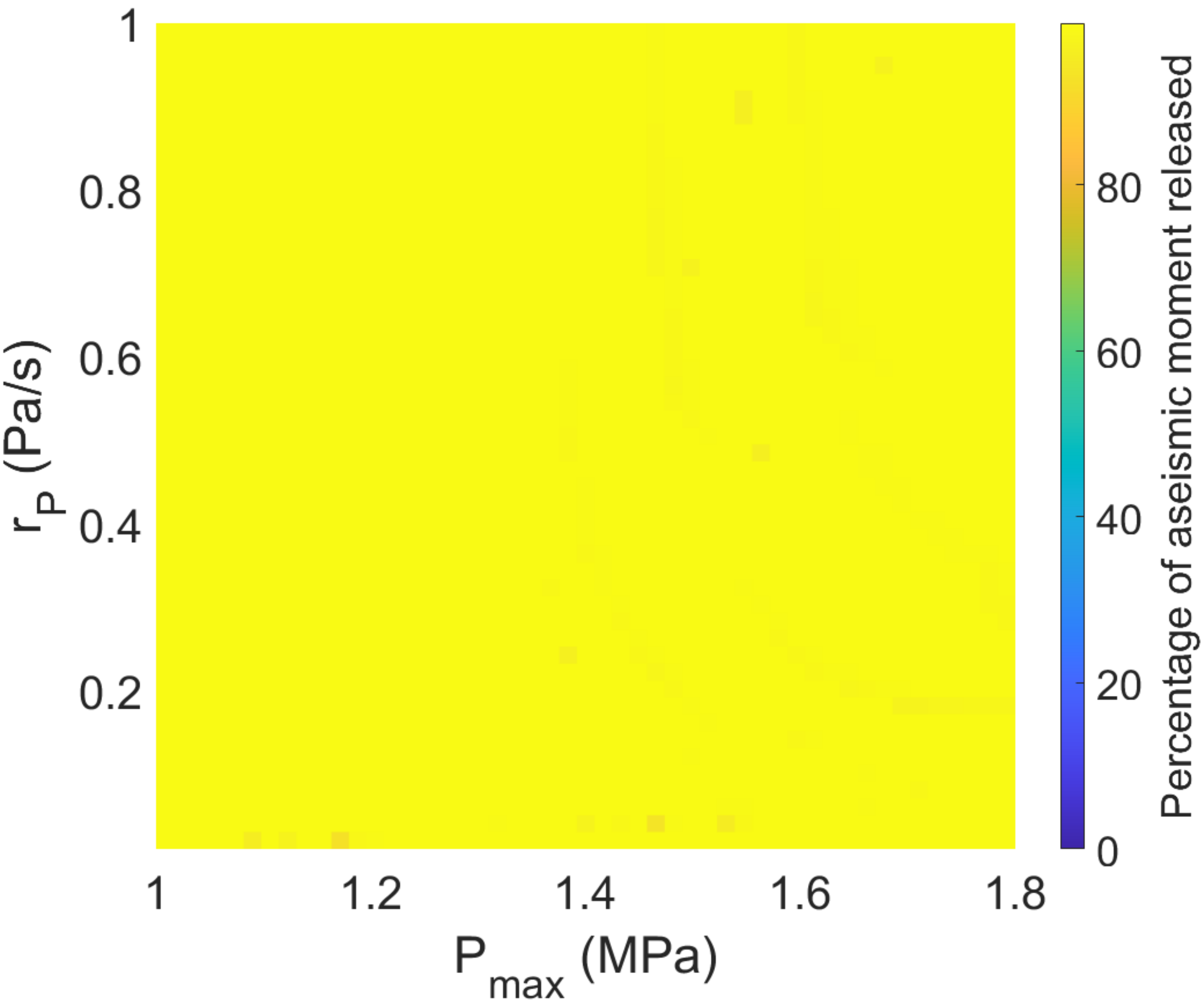

Supplementary Figure 4: Percentage of aseismic energy released relative to the total energy released. Cumulative aseismic moment released during perturbation as a percentage of the total moment released (seismic moment + aseismic moment). In all cases, aseismic energy accounts for over 90% of the total energy released.

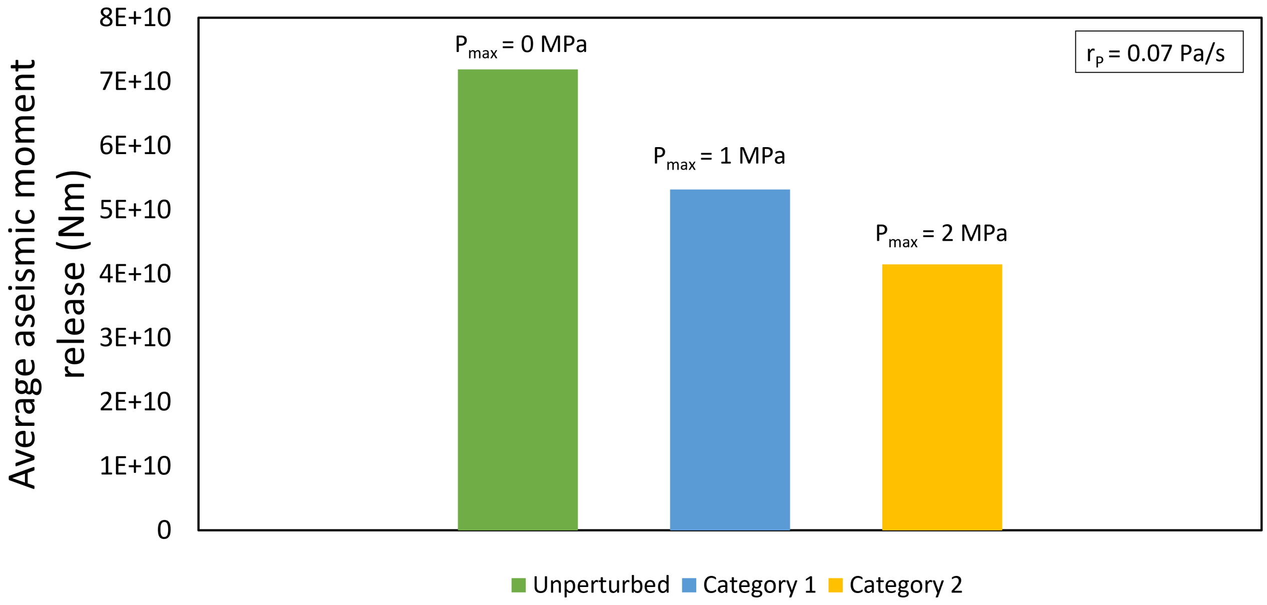

Supplementary Figure 5: Average aseismic moment release for different scenarios. Average aseismic moment released on the fault for 40 years starting one year after the end of the perturbation for different categories of perturbation. In all cases, the aseismic moment released after the perturbation ends is lower than that released during the same duration in the unperturbed case

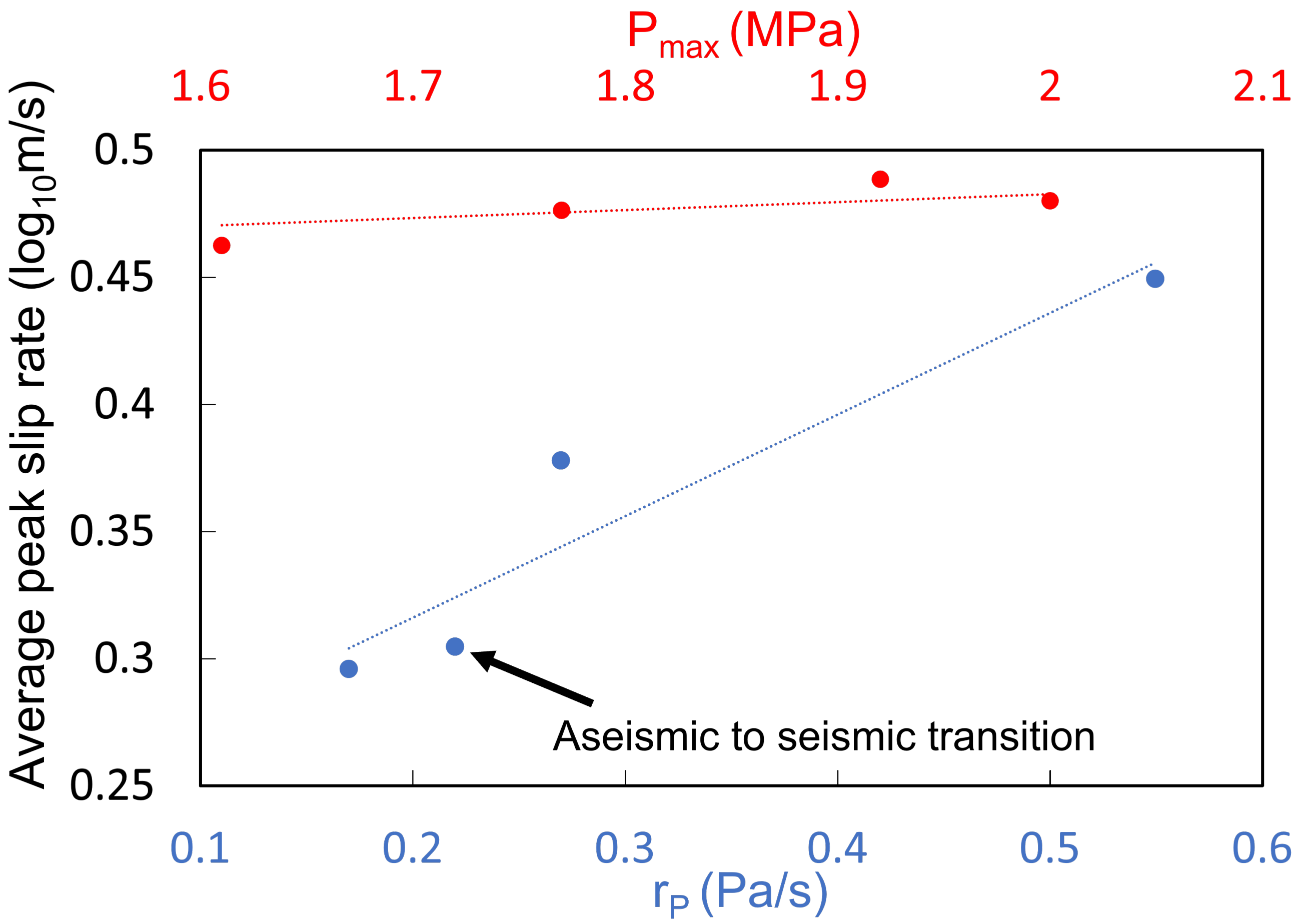

Supplementary Figure 6: Evolution of average peak slip rate as r_P and P_{max} are increased. $\log_{10}$ of the average peak slip rate of all triggered transients and earthquakes, versus r_P (blue) and P_{max} (red), showing the evolution of the triggered cluster. The point at which the first triggered fault response transitions from an aseismic transient to a seismic event is marked with an arrow. r_P = 0.07 Pa/s when P_{max} is varied and P_{max} = 1.7 when r_P is varied.
